# Inferring the Urban Transmission Potential of Bat Influenza Viruses

**DOI:** 10.3389/fcimb.2020.00264

**Published:** 2020-06-03

**Authors:** Efstathios S. Giotis

**Affiliations:** ^1^Section of Molecular Virology, Department of Infectious Diseases, Imperial College London, London, United Kingdom; ^2^School of Life Sciences, University of Essex, Colchester, United Kingdom

**Keywords:** bats, influenza virus, haemagglutinin, neuraminidase, Major Histocompatibility Complex (MHC) class II, sialic acids

## Abstract

Bats are considered natural reservoirs of various, potentially zoonotic viruses, exemplified by the influenza A-like viruses H17N10 and H18N11 in asymptomatic Neotropical bats. These influenza viruses are evolutionarily distinct, are poorly adapted to laboratory mice and ferrets and cannot reassort *in vitro* with conventional strains to form new influenza subtypes. However, they have attracted renewed attention following reports that their entry in host cells is mediated by the trans-species conserved MHC-II proteins, suggesting that they hold zoonotic potential. Despite the recent studies, the viruses' epidemiology and public health significance remain incompletely understood. Delineating the mechanistic basis of the interactions with their hosts and assessing their global distribution are essential in order to fully assess the zoonotic threat that these strains pose.

## Introduction

**Figure 1:**
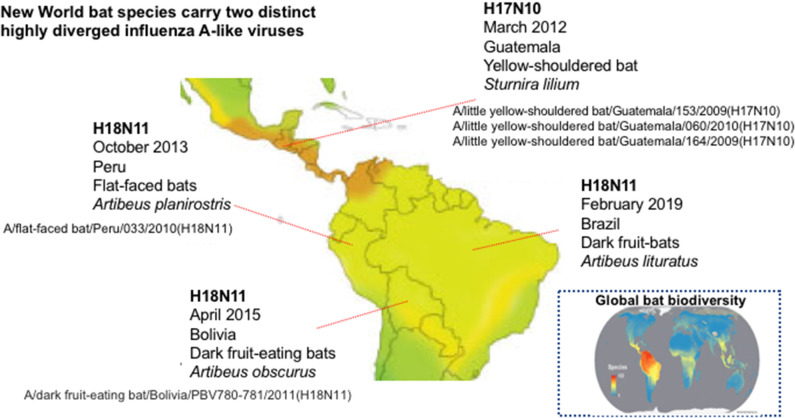
Countries of Central and South America where bat influenza A viruses have been reported. Map showing the global pattern of bat species richness was provided by Clinton Jenkins (see BiodiversityMapping.org) using species data from The IUCN Red List of Threatened Species (2018; https://www.iucnredlist.org) (Jenkins et al., 2013).

The Severe Acute Respiratory Syndrome (SARS), Middle East Respiratory Syndrome (MERS), Nipah (NiV), Hendra (HeV), and Ebola (EBOV) viruses' outbreaks confirmed the inextricable nature of human and bat health and disease and highlighted that focusing on the “spillover” potential of known, and novel, bat viruses is critical to predict and prevent pandemics. The remarkable ability of bats to coexist with a wide range of viruses that would be pathogenic in flightless mammals (Fl^−^M) is not yet fully understood but possibly relates to their unique, flight-adapted antiviral immunity (Calisher et al., 2006; Hayman et al., 2013). Intriguingly, genetic material from viruses that resemble influenza type A viruses has been recovered from asymptomatic fruit bats of the Neotropic bat family *Phyllostomidae* (*Sturnira lilium* and *Artibeus planirostris*) in several countries of Central and South America (Figure 1) (Tong et al., 2012, 2013; Campos et al., 2019). Influenza A viruses (IAVs) are orthomyxoviruses with eight single-stranded negative-sense viral RNAs (vRNAs) encapsidated into viral ribonucleoproteins (vRNPs). IAVs emerge from aquatic birds, *via* genome reassortment and mutation, and are able to cause epidemics (and sporadic pandemics; Simonsen, 1999) in humans, lower animals and birds (Simonsen, 1999). The bat influenza viruses (BatIVs) are phylogenetically distinct from the conventional IAVs and they were designated as H17N10 and H18N11 (Table 1). Bats in Latin America, but not in Central Europe, have been found seropositive for BatIVs (Tong et al., 2013; Fereidouni et al., 2015). Antibodies against human H2N2 and H3N2, as well as classical H9, have also been found in bats elsewhere (L'vov et al., 1979; Kelkar et al., 1981; Isaeva et al., 1982), suggesting they are susceptible to IAV infection. The epithelial kidney cells of flying foxes (*Pteropus alecto*) co-express both avian (α2,3-Gal) and human (α2,6-Gal) sialic acid (SA) receptors (Chothe et al., 2017) and are thus susceptible to infection by both avian and human IAVs, but more importantly they allow reassortment between co-infecting influenza viruses (Dlugolenski et al., 2013). The N-terminal domain of the H17N10 PA subunit of the influenza virus polymerase complex possesses endonuclease activity comparable to that of IAVs (Tefsen et al., 2014). Equally intriguingly, in the position 627 of the polymerase gene PB2, one of the most commonly identified IAV virulence markers, BatIVs have a serine compared to glutamic acid in avian and lysine in mammalian influenza strains, suggestive of an alternative evolutionary pathway for avian IAV's adaptation in mammals (Mehle, 2014). This all raised the question whether novel IAVs could emerge from bats to which human and animal populations would be immunologically “naïve,” causing pandemics.

**Table 1:** List of distinctive features of IAVs and BatIVs.

	**IAVs**	**BatIVs**
Known hosts	Birds, humans, swine, equine, and marine mammals	New World bats
Clinical manifestation	Mild to severe respiratory disease to humans and birds, cause outbreaks/epidemics, and sporadically pandemics	Asymptomatic (unclear)
Cell surface receptors/entry factors	Sialic acids	MHC-II
Role of haemagglutinin	Cell attachment/entry	Cell entry
Role of neuraminidase	Sialidase activity	Unknown
Culture in embryonated chicken eggs	Yes (usually)	No
Agglutination of red blood cells	Yes (usually)	No
Genetic drift	Yes	Yes
Genetic reassortment	Yes	Yes (not with IAVs)
Main transmission routes	Respiratory droplets, direct contact/fecal-oral	Fecal-oral (unclear)

## BatIVs are Distinct and Employ Unconventional Receptors for Cell Entry

The genetic material of BatIVs is similar to classic flu viruses, but their surface glycoproteins haemagglutinin (HA) and neuraminidase (NA) are evolutionarily and functionally diverged (Li et al., 2012; Zhu et al., 2012; Sun et al., 2013; Tong et al., 2013). BatIVs cannot be cultured in embryonated chicken eggs and do not agglutinate red blood cells (Tong et al., 2012, 2013). Initial efforts by researchers to isolate live infectious BatIVs directly from bats failed, due to unavailability of permissive cell lines (Ciminski et al., 2017). In addition, research on these viruses was further complicated by the dearth of bat cell lines and the limited bat genomic data. Attempts to circumvent these limitations have included: (i) using HA-17 or HA-18 pseudotyped vesicular stomatitis virus (VSV) and HIV-1 based lentiviruses (Hoffmann et al., 2016; Maruyama et al., 2016; Carnell et al., 2018; Giotis et al., 2019), (ii) engineering BatIV/IAV chimaeric viruses (Juozapaitis et al., 2014; Zhou et al., 2014), and (iii) reconstructing authentic BatIVs using reverse genetics (Moreira et al., 2016; Sato et al., 2019; Zhong et al., 2019). HA17-VSV was able to infect bat cell lines (EidNi, HypNi, and EpoNi) but only a few of the common Fl^−^M cell lines, including human U-87 MG glioblastoma and SK-Mel-28 melanoma cells, canine RIE 1495 and MDCK II kidney cells (Hoffmann et al., 2016; Maruyama et al., 2016; Moreira et al., 2016). The identification of MDCK II, in particular, as susceptible cell lines to BatIVs opened the way for a more comprehensive characterization of these strains (Moreira et al., 2016; Giotis et al., 2019; Karakus et al., 2019).

Crystal structure analyses revealed that the bat haemagglutinins display typical HA protein folds but lack any obvious cavity to accommodate SA, which are the conventional receptors of IAVs. Recently, two independent studies demonstrated that the cell-entry of H17N10 (Giotis et al., 2019) and H18N11 (Karakus et al., 2019) is mediated by MHC-II receptors that are well-conserved in many species. Hence, immortalized cell lines that express MHC-II receptors on their surface such as several human leukemia and lymphoma cell lines Raji, Ramos, and BJAB B-lymphocytes could be used for the study of the viruses' biology (Giotis et al., 2019). Interestingly, ectopic expression of pig, mice, and chicken MHC-II have been shown to confer susceptibility to H18N11 in non-susceptible cells (Karakus et al., 2019) implying a potential role for the respective animals as intermediary hosts. It is as yet unclear whether MHC-II receptors function with other unknown factors to facilitate virus internalization and also whether the viruses remain cell associated following cell-entry and are being passed on by direct cell-cell contact.

MHC-II molecules occur as three highly polymorphic isotypes (HLA-DR, HLA-DP, and HLA-DQ). They are selectively expressed on the surface of professional antigen presenting cells (APCs), act as ligands for the T-cell receptor (TCR), and play a key role in the presentation of foreign antigens to CD4^+^ T helper cells and immune surveillance (Jones et al., 2006; Roche and Furuta, 2015). Bats contain all classical MHC class II gene families that are responsible for antigen presentation with an extra *DRB2* gene copy located outside the MHC-II region in *P. alecto* (Ng et al., 2017). The MHC-II dependent cell entry suggests that BatIVs might hijack APCs such as B lymphocytes and dendritic cells for viral dissemination and/or survival perhaps in the early stages of infection. It is unknown to what degree their binding to APCs might influence the global outcome of the host immune responses. A blockade of TCR recognition by steric hindrance as described for Epstein-Barr virus (Ressing et al., 2003, 2005; Wiertz et al., 2007), could explain the asymptomatic status of the infection in the captured New World bats.

## The Enigmatic Role of bat Influenza Viruses' Neuraminidases

The bat neuraminidases (NAs) are structurally similar to classical NAs but lack conserved amino acids for SA binding or cleavage (Li et al., 2012; Zhu et al., 2012). Moreover, unlike classical NAs, they display no enzymatic activity (Garcia-Sastre, 2012; Carnell et al., 2018), have a dispensable role in viral entry (Hoffmann et al., 2016; Maruyama et al., 2016; Giotis et al., 2019) and their function is not yet elucidated. Recent studies demonstrated that the passage of reverse-genetics-generated H18N11 virus in cell cultures, accumulates mutations in the N11 protein that increase virus titers in culture and may enhance organ tropism *in vivo* (Zhong et al., 2019). Ciminski et al. demonstrated by utilizing a chimeric bat influenza virus (PR8-H18N11) that viruses encoding the full-length N11 protein exhibited a growth advantage over viruses that encode a truncated protein version and also showed that N11 is essential for viral transmission (Ciminski et al., 2019b). Another study showed that the N10 protein facilitates heterosubtypic (H5 and H7) influenza hemagglutinin-bearing pseudotype release in the absence of another source of neuraminidase, indicating a possible role of N10 in viral release (Carnell et al., 2018). It has been proposed that N11 downregulates MHC-II, thereby facilitating virion release but mechanistic data for such function is as yet missing (Ciminski et al., 2019b). There is no evidence that a functional balance exists between bat HAs and NAs although it is possible that the proteins coordinate their actions. Despite the recent progress in our understanding, the exact function of bat NAs remains an enticing mystery.

## Intraspecies and Interspecies Transmission of bat Influenza Viruses

It is now becoming more evident that BatIVs may transmit in a different manner than conventional flu strains. It has recently been shown that the H18N11 virus readily transmits between bats (Ciminski et al., 2019b). Following experimental intranasal H18N11 infection, the Neotropical *Phylostomidae* bat species *Artibeus jamaicensis* shed high viral loads via the fecal route and were able to infect naïve contact animals (Ciminski et al., 2019b). Histopathological analysis of the infected bats indicated that viral replication proceeds in the follicle-associated epithelium of gut-associated lymphoid tissue, suggesting virus uptake from the gastrointestinal lumen (Ciminski et al., 2019b), in line with the rich gut epithelial expression of MHC-II (Wosen et al., 2018). Furthermore, the researchers detected high loads of H18N11 viral transcripts in rectal swabs and excretions (Ciminski et al., 2019b). Collectively, these findings imply that an environmental (fecal-oral) mode of BatIVs transmission is more likely than an airborne one, albeit the latter is not yet compellingly disproved. Other infection routes including subcutaneous, transplacental, vaginal, intracranial infections have not yet been reported. In contrast to bats, H18N11 has been reported to have limited replicative ability in laboratory mice and more interestingly in ferrets which share similar lung physiology and SA distributions to humans (Ciminski et al., 2019b; Karakus et al., 2019; Zhong et al., 2019). Whether these observations are animal/infection-route-dependent or they actually reflect a low zoonotic risk for BatIVs remains to be seen.

In absence of conclusive scientific proof, the question remains as to whether BatIVs are confined to a sylvatic transmission cycle and perpetuate in Neotropical bat populations or are capable of urban adaptation. Anthropogenic disruptions of ecological habitats that led to urban transmission of other enzootic bat viruses (i.e., HeV, NiV) have been extensively described in the scientific literature. Despite the increasing disturbances in the fire-prone Neotropical forests, New World bats have not yet been implicated in the transmission of zoonotic viruses, other than rabies, to humans (Moratelli and Calisher, 2015). Even so, Latin America is home to the richest and most diverse bat fauna in the world (Figure 1) including almost 150 *Phyllostomidae* species (Jenkins et al., 2013) in which BatIVs have been detected. Unlike African and Asian bats which are consumed regularly, New World bats are only eaten by few native indigenous people (Moratelli and Calisher, 2015). A possible exposure to infected bat blood and body fluids may hypothetically create a pathway for disease transmission to humans. Another scenario may involve the accidental introduction of BatIVs to the local fauna or other *Phyllostomidae* species such as the widespread blood-eating vampire bat (*Desmodus rotundus*), which thrives in both native and anthropogenically transformed ecosystems (Bergner et al., 2020). Vampire bats have long been suspected of passing on rabies to humans and livestock in Latin America by biting and scratching (Rupprecht et al., 2002). It will be useful to explore in future studies whether haematophagous bats can act as maintenance hosts for BatIVs and if their biting can form a potential zoonotic transmission route either directly or through mammalian intermediate hosts.

## The Host Factors Regulating BatIVs Replication are Poorly Understood

BatIVs, like all viruses, have to compromise with positive and negative genetic factors present in target cells for their survival at each replication stage. Little is known regarding the interaction of BatIV proteins and RNA with the host or viral factors even though such interactions may determine the fate and/or efficiency of infection, transmission, and epidemic potential of the viruses. Previous studies revealed that the bat Nonstructural NS1 proteins can act as interferon (IFN) antagonists in human cells, and likely inhibit induction of IFN at a pretranscriptional level (Ciminski et al., 2017). More recently, it has been shown that the IFN-induced human MxA protein controls the replication of H18N11, but it is not clear whether sufficient MxA-escape mutations in H18N11 NP can be acquired *in vivo* that could potentially result in full MxA resistance (Ciminski et al., 2019a). Nearly all lab work examining host and viral immune-modulating proteins is performed with human/rodent cell lines. The difficulty in interpreting these data is that evolutionarily-optimized immune factors behave differently in non-natural hosts. Certainly, comprehensive kinetic analyses of immune-responses to BatIVs using primary or immortalized bat cell lines will be particularly informative. For instance, a comparison of the transcriptome of BatIV infected versus uninfected bat cells could help us identify specific immune genes contributing to host resistance and the molecular mechanisms underlying the viral pathogenesis.

## Do bat Influenza Viruses Pose a Zoonotic Risk?

Reassortment of gene segments between co-infecting viruses is a key process mediating the genetic evolution of influenza viruses and the generation of novel epidemic and pandemic strains. BatIVs are able to reassort between themselves but not with conventional IAVs *in vitro* (Juozapaitis et al., 2014; Zhou et al., 2014). Generation of bat chimeric viruses was only possible when the HA/NA coding regions were flanked with the authentic BatIV packaging signals demonstrating packaging incompatibilities between IAVs and BatIVs (Juozapaitis et al., 2014; Zhou et al., 2014). This finding dismisses the scenario of emergence of a new “reassortant” virus with human/avian IAVs unless the bat viruses undergo major genetic changes over time.

However, the abilities of BatIVs to (i) reassort between themselves, (ii) to mutate in order to infect and transmit sustainably among their hosts, and (iii) enter human HLA-DR^+^ cells, highlight that a zoonotic transmission of BatIVs is theoretically possible. The documented spillover of other non-reassortant bat-borne RNA viruses following continued host-pathogen interaction (i.e., NiV and EBOV) lends certain credence to this hypothesis, albeit clearly, supporting evidence is lacking.

To explore the ecological and evolutionary dynamics of these and possibly other unknown influenza-A-like viruses, further prevalence and serological studies in Neotropical bat populations are required coupled with the surveillance of bat-exposed humans and livestock. Surveys of bat colonies have previously led to identification of other zoonotic viruses, including HeV in Pteropus sp. in Australia, NiV in Pteropus lylei in Thailand, and MARV in Rousettus aegyptiacus in Uganda (Wacharapluesadee et al., 2010; Amman et al., 2012; Field et al., 2015). A computational study which used spatial empirical models to trace the steps of emergence of bat viruses and the transmission opportunities to humans pinpointed sub-Saharan Africa as the top-priority location for pathogen discovery in wildlife (Brierley et al., 2016). West Subsaharan Africa, in particular, hosts enormous populations of sedentary and migrating bats living in proximity to human and animal populations. Considering the number, the morbidity and mortality of emerging viruses that are hosted in African bats as well as the serological evidence against IAVs (Freidl et al., 2015), future surveillance and serological studies could lead to the identification of novel bat influenza subtypes.

## Conclusions

In summary, BatIVs are unconventional influenza viruses that resemble to some extent more paramyxoviruses rather than typical orthomyxoviruses. Despite the recent findings on the cell entry factors and NAs of these viruses, it is clear that we only scratched the surface in terms of characterization of these viruses. The scientific evidence so far indicate a limited spillover risk but data is not conclusive enough to dismiss out of hand the possibility of zoonotic transmission. Forecasting viral spillover is a challenging task and additional interdisciplinary and more up-to-date approaches are warranted to fully appreciate the ecology and the implications of these viruses for public health. Future studies on BatIVs hold extra value as they can provide broader mechanistic insights into the molecular biology of influenza viruses and might inform translational studies.

## Author Contributions

The author confirms being the sole contributor of this work and has approved it for publication.

## Conflict of Interest

The author declares that the research was conducted in the absence of any commercial or financial relationships that could be construed as a potential conflict of interest.
